# Respiratory Sinus Arrhythmia Mediates the Relation Between “Specific Math Anxiety” and Arithmetic Speed

**DOI:** 10.3389/fpsyg.2021.615601

**Published:** 2021-02-19

**Authors:** Jiuqing Tang, Yun Su, Yu'e Yao, Hugo Peyre, Ava Guez, Jingjing Zhao

**Affiliations:** ^1^School of Psychology, Shaanxi Normal University, Shaanxi Provincial Key Research Center of Child Mental and Behavioral Health, Xi'an, China; ^2^Laboratoire de Sciences Cognitives et Psycholinguistique (ENS, EHESS, CNRS), Ecole Normale Supérieure, PSL University, Paris, France; ^3^Neurodiderot, INSERM UMR 1141, Paris Diderot University, Paris, France; ^4^Department of Child and Adolescent Psychiatry, Robert Debre Hospital, APHP, Paris, France

**Keywords:** math anxiety, physiology, arithmetic, RSA, affective

## Abstract

There is a growing consensus that math anxiety highly correlates with trait anxiety and that the emotional component elicited by math anxiety affects math performance. Yet few studies have examined the impact of “specific math anxiety” (high math anxiety and low other kinds of anxiety) on math performance and the underlying physiological and affective mechanism. The present study examines the mediation effect of heart rate variability—an affective measurement indexed by respiratory sinus arrhythmia (RSA)—in the relationship between specific math anxiety and arithmetic speed. A total of 386 junior high school students completed a self-reported questionnaire to measure their anxiety level. Among this sample, 29 individuals with specific math anxiety (high math anxiety and low reading and trait anxiety), 29 with specific reading anxiety (high reading anxiety and low math and trait anxiety), 24 with specific trait anxiety (high trait anxiety and low math and reading anxiety), and 22 controls (low math, trait and reading anxiety) were selected to participate in an arithmetic task and a reading task while RSA was recorded when they performed the tasks. Results revealed that individuals with specific math anxiety showed lower RSA and longer reaction time than the other three groups in the arithmetic task. Regression and mediation analyses further revealed that RSA mediated the relation between specific math anxiety and arithmetic speed. The present study provides the first account of evidence for the affective hypothesis of specific math anxiety and suggests that affective responses may be an important mechanism underlying the detrimental effect of specific math anxiety on math performance.

## Introduction

Individual differences in mathematic achievement have been the focus of a wide strand of research and a growing area of concern for policy makers and educational psychologists in the last decades. Although several factors have been shown to explain individual differences in mathematic achievement (such as socioeconomic status, see Ritchie and Bates, 2013), math anxiety has been widely accepted to have a robust negative impact on math performance (Hill et al., 2016; Sorvo et al., 2019; Xie et al., 2019; Zhang et al., 2019).

Math anxiety typically refers to the emotional response of tension and anxiety in thinking about or engaging in situations related to mathematical tasks (Hembree, 1990). It has been found to correlate with a more general anxiety, namely, trait anxiety (e.g., Hill et al., 2016). Trait anxiety is a relatively stable emotional state, in which people tend to perceive external stimuli as danger or threat (Spielberger, 1983). Trait anxiety has also been identified to have negative effects on performance in math tasks (Owens et al., 2008). This might be because math anxiety and trait anxiety had shared genetic and environmental risk factors, suggested by a behavioral genetic twin study (Wang et al., 2014). Yet the effect of math anxiety on math performance remained significant when the influence of trait anxiety was regressed out, but the effect of trait anxiety on math performance was not significant when math anxiety was regressed out (Justicia-Galiano et al., 2017). This has led to a speculation that children with high trait anxiety are more likely to develop math anxiety later; and once children developed math anxiety, math anxiety would become a better predictor of math performance than trait anxiety (Justicia-Galiano et al., 2017). In other words, math anxiety may influence math performance independently from trait anxiety in teenagers who already develop math anxiety. Nevertheless, few studies examined this hypothesis directly. The current study for the first time tests the impact of “specific math anxiety” (high math anxiety but low trait anxiety) on math performance in middle school adolescents.

In terms of the underlying mechanism explaining the relation between math anxiety and math performance, two different dimensions in math anxiety scales have been identified to play important roles: the cognitive dimension and the affective dimension (Wigfield and Meece, 1988; Ho et al., 2000). Cognitive dimension of math anxiety refers to worrisome thoughts about one's math performance, while affective dimension of math anxiety represents nervous or tense feelings and unpleasant physical reactions in mathematical situations (Namkung et al., 2019). A recent meta-analysis found that math performance was related to both cognitive and affective dimensions of math anxiety (see meta-analysis by Namkung et al., 2019).

On the one hand, the cognitive interference theory, also named the debilitating anxiety hypothesis, has been developed regarding how the cognitive dimension of math anxiety impacts mathematic performance (Carey et al., 2016). The theory argues that math anxiety interferes with cognitive processes, which in turn leads to poor math performance (Morsanyi et al., 2014). The interference mechanism could lie in information encoding, processing, and retrieving processes. During the information encoding process, high-math-anxiety individuals tend to avoid math-related situations, thus leading to less training in math (Hirvonen et al., 2012). During information processing and retrieving processes, high-math-anxiety individuals' cognitive resources work like a dual task setting in math tasks: one for anxiety and the other for the task (Ashcraft and Krause, 2007). Thus, limited working memory in these individuals results in slower reaction time (RT) and higher error rates in math tasks. A number of studies have provided experimental support to the cognitive hypothesis of math anxiety on math task performance (see meta-analysis by Namkung et al., 2019). For example, complex and difficult math problems that require more cognitive processing and occupy more working memory and attentional resources typically elicited higher math anxiety. Hunt et al. (2017) found that participants with math anxiety responded slower and produced more error responses in three-digit arithmetic problems than two-digit arithmetic problems, and their blood pressure only significantly increased for three-digit arithmetic problems but not for two-digit arithmetic problems. An event-related potential (ERP) study also reported that high-math-anxiety individuals made more errors and showed a larger P2 component in math calculation, indicating they invested more attentional resources while performing math tasks (Núñez-Peña and Suárez-Pellicioni, 2015).

On the other hand, there is a growing consensus that emotional component induced by math anxiety may also affect math performance. For instance, recent studies on the central nervous system (CNS) have found higher activation of brain areas associated with pain perception and negative emotional process in math anxiety individuals before and during math tasks (Lyons and Beilock, 2012; Young et al., 2012). Besides, physiological responses related to anxiety such as increases in heart rate (Ushiyama et al., 1991), secretory immunoglobulin A (sIgA) concentration and secretion rate (Willemsen et al., 2000), higher cortisol level (see review by Chang and Beilock, 2016), and change in systolic blood pressure (Hunt et al., 2017) have been found in individuals with math anxiety when they performed math tasks. These studies indicate CNS reflected by brain activation, hypothalamic–pituitary–adrenal (HPA) axis reflected by cortisol, and the mixture of sympathetic nervous system and parasympathetic nervous system reflected by heart rate and blood pressure participated in emotional process of math anxiety. Apart from the physiological indicators mentioned above, respiratory sinus arrhythmia (RSA) is a real-time response solely innervated by the parasympathetic nervous system (PNS) that captures unique emotional reactions other than cortisol, heart rate, and blood pressure (Frazier et al., 2004). Examining RSA might provide new insight into understanding the role of the parasympathetic nervous system in math-anxious individuals.

RSA, which refers to the natural log equivalent of the high-frequency component (0.15–0.5 Hz) of heart rate variability (HF-HRV), is the rhythmic fluctuation of cardiac interbeat intervals (IBIs) during breathing and reflects the control of the parasympathetic nervous system entirely mediated by vagal tone (Berntson et al., 1993; Porges, 1995, 2007). RSA in the resting stage is a predictor of both concurrently and prospectively affective responses to stress (Bornas et al., 2005; Friedman, 2007). Some studies revealed that RSA reactivity, that is, changes of RSA in individuals exposed to external stimulus from RSA in the resting state, could also reflect emotion changes (Gentzler et al., 2009; Fortunato et al., 2013; Utendale et al., 2014). According to polyvagal theory, RSA suppression (RSA reactivity decreases from baseline) is an adaptive response to stressors, while RSA augmentation (RSA reactivity increases from baseline) indicates successful emotion regulation (Porges, 1995). Numerous studies indicated that individuals with anxiety disorders tend to show lower RSA in resting state and reactivity than controls (Cohen et al., 1998; Jovanovic et al., 2009; Blom et al., 2010). Moreover, the amount of RSA suppression positively correlated with anxiety levels (Fortunato et al., 2013; Graziano and Derefinko, 2013). Therefore, RSA suppression in individuals with specific math anxiety might be larger compared with individuals without specific math anxiety when they perform math tasks. RSA suppression might also play a link between specific math anxiety and performance in math tasks. Yet this hypothesis has never been tested.

In sum, the main purpose of the present study was twofold. First, we aimed to examine whether individuals with specific math anxiety (high math anxiety and low other kinds of anxiety) showed worse performance and larger RSA suppression in math tasks than individuals with relatively low math anxiety, especially those with high trait anxiety. Second, we aimed to explore the mediation effect of RSA in the relationship between specific math anxiety and performance in math tasks. We predicted that if the affective component of math anxiety played a role in math performance, larger RSA suppression should be observed in individuals with specific math anxiety in math-related situations, and physiological response (indexed by RSA suppression) should mediate the relation between specific math anxiety and math performance.

## Materials and Methods

### Participants

A total of 386 students from the seventh grade (235 students) and eighth grade (151 students) in a junior middle school of Xi'an participated in this study. All students were native Chinese speakers with normal vision and hearing abilities. Each student's parental education was recorded as the highest diploma obtained, coded on a 1–8 scale (1: primary school, 2: junior school, 3: senior school, 4: junior college education, 5: university, 6: master, 7: doctor, 8: post-doctoral). Ethics approval for the study was granted by Shaanxi Normal University. The study was consented by all the participants, their parents, and their teachers. A total of 104 students selected from these 386 students participated in math and reading tasks while their RSAs were recorded.

### Measures and Procedure

#### Screening Phase

All 386 students participated in the screening phase. Math anxiety, reading anxiety, and trait anxiety scales were administrated to each of these students. The purpose of including reading and trait anxiety in this study was to guarantee that students with specific math anxiety were those with low reading and trait anxiety.

#### Math Anxiety

Mathematics Anxiety Rating Scale (R-MARS) was used to measure math anxiety (Plake and Parker, 1982). A total of 21 items were included in the scale with a 1–5 Likert scale from 1 (not anxious) to 5 (very anxious). The participants needed to rate the degree of their anxiety affect in each situation. Scores ranged from 21 to 105. Higher summed scores indicate higher math anxiety. Cronbach's α for this scale is 0.92.

#### Reading Anxiety

A Chinese reading anxiety scale revised based on the Foreign Language Reading Anxiety Scale (FLRAS) (Saito and Samimy, 1996) was adopted to measure reading anxiety. Participants answered the 20 items on a 1–5 Likert scale, with a higher score indicating more anxiety. The score ranged from 20 to 100. The scales' reliability and validity were good (Cronbach's α = 0.87).

#### Trait Anxiety

A subscale of the trait anxiety inventory (T-AI) from the revised State–Trait Anxiety Inventory (STAI) was used to measure the trait anxiety of each student (Spielberger, 1983). Trait anxiety refers to an individual's anxious or worrying dispositions in general (Spence, 1997). Participants indicated how often they experienced the situations illustrated in the statements such as “I feel happy” and “I lack self-confidence” using a 1–4 Likert scale with 1 labeled “not at all” and 4 labeled “almost always.” There were 20 items in total. The score ranged from 20 to 80. This scale has adequate internal consistency (Cronbach's α = 0.82).

#### Testing Phase

Due to the limitation of resources in collecting RSA data and given the high correlations between math anxiety and trait/reading anxiety, 104 participants from the total sample were selected to participate in the testing phase. These selected participants were divided into four groups: “specific math anxiety” group, “specific trait anxiety” group, “specific reading anxiety” group, and “almost no anxiety” group. Twenty-nine students (15 boys and 14 girls) whose math anxiety scores were above the 75% percentile while trait anxiety and reading anxiety scores were below the 25% percentile were included into the math anxiety group. Twenty-nine students (19 boys, 10 girls) whose reading anxiety scores were above the 75% percentile while trait anxiety and math anxiety scores were below the 25% percentile were included into the reading anxiety group. Twenty-four students (14 boys, 10 girls) whose trait anxiety scores were above the 75% percentile while math anxiety and reading anxiety scores were below 25% percentile were included into the trait anxiety group. Finally, 22 students (14 boys and 8 girls) whose trait anxiety, math anxiety, and reading anxiety scores were all below the 25% percentile were selected as no-anxiety controls. Sex and age were matched across the four different groups. Math anxiety, trait anxiety, and reading anxiety were normally distributed in this selected sample (see Supplementary Table S1). Participants completed two tasks (math task and reading task) while their RSAs were recorded. The order of the two tasks was counterbalanced among participants, and participants did not know about the task order until they started the tasks. Each participant was individually tested.

#### Math Task—Arithmetic

The math task was created using the Python software and included four types of one-digit arithmetic operations: additions, subtractions, multiplications, and divisions. For example, “1+9=.” Each operation consisted of 20 trials. There were 80 trials in total. Problems were presented in size 72, black Arial style font. The operations were randomly presented on the computer screen with one operation per presentation. In each presentation, two answer options were provided below each operation on the computer screen, with one number on the left side and the other on the right side. Participants were asked to press F (if the number on the left side is correct) or J (if the number on the right side is correct) on the keyboard as quickly and as accurately as possible to choose the correct answer when they saw the numbers and operations on the computer screen. Reaction time (RT) was recorded and measured from the onset of the presentation of the numbers and operation in each trial until participants made a response. Before formal experiment, each individual performed a practical block to familiarize the protocol. Response accuracy and RT of each individual were recorded via a computer.

#### Reading Task—Character Recognition

The reading task required each participant to read aloud Chinese characters in a list one by one as quickly and as accurately as possible. The character list included 195 Chinese characters that were selected from Chinese middle school textbooks with increased difficulty. The characters were listed in 13 pages with each page having 15 characters. Accuracy and total naming time of each individual were recorded. The reading task served as a control task in this study.

#### Heart Rate Variability

SOMNOtouch RESP (SOMNOmedics, Germany) was used to collect electrocardiogram (ECG) data at a sampling rate of 256 Hz during the completion of math and reading tasks of each participant, with electrodes placed on each participant's ribs and clavicles. R-waves were identified in DOMINO light software 1.4.0 (SOMNOmedics, Germany) automatically and by a researcher manually and then processed in the Interbeat Interval (IBI) Analysis System. Data with artifacts were detected and excluded from the analysis. The R–R intervals required for spectral analysis was obtained by linear interpolation at 4 Hz. Then data were further detrended by a smoothness prior approach (Tarvainen et al., 2002). IBI power spectra were generated by an autoregressive algorithm (Tarvainen et al., 2002). RSA was computed by the logarithm value of the high-frequency IBI power spectra. The RSA baseline was recorded 3 min before the start of each task. RSA reactivity was defined as “RSA in task – RSA baseline.”

### Data Analysis

Group differences in subject characteristics, anxiety, physiological, and behavioral measures were tested through chi-square or general linear models with accuracy, RT, naming time, RSA, and self-reported anxiety level as dependent variables and group (math anxiety vs. trait anxiety vs. reading anxiety vs. control) as a between-subject variable. Results were corrected for multiple comparisons of groups using the false discovery rate (FDR) correction. As previous studies have found that girls tended to report higher math anxiety than boys (Goetz et al., 2013; Hill et al., 2016; Sokolowski et al., 2019; Sorvo et al., 2019; Xie et al., 2019) and we also observed gender differences in our sample (Supplementary Table S2), we included gender as a covariate in all analyses.

A correlative analysis between anxiety, RSA measurements, and behavioral task performance was performed. Then significant correlations between math anxiety, RSA, and arithmetic RT were further examined using hierarchical linear regression analysis and mediation analysis. To examine the effects of “specific math anxiety” group membership on RSA and arithmetic RT, “specific trait anxiety” group, “specific reading anxiety” group, and “almost no anxiety” group were merged as a non-specific math anxiety group. For hierarchical linear regression analysis, arithmetic RT was entered into the model as a dependent variable. Gender and reading-related measurements were entered into the model as covariates in the first step (step 1); then group membership was entered into the model as an independent variable in the second step, followed by RSA in the third step. For mediation analysis, three path model analyses were run with PROCESS macro v.2.15 in SPSS. In the first path model analysis, “specific math anxiety” group membership with two groups (specific math anxiety group coded as 1 and non-specific math anxiety group coded as 0) was entered into the model as an independent variable, while RSA was entered into the model as a mediator, arithmetic RT as a dependent variable, gender and reading-related measures as covariates, respectively. In the second and third path model analyses, group membership with four groups was coded as dummy variables and was entered into the model as an independent variable. In the second path model, the specific math anxiety group was coded as a reference group (coded as 000), while no-anxiety control group (coded as 100), specific reading anxiety group (coded as 010), and specific trait anxiety group (coded as 001) were coded as experimental groups. In the third path model, the no-anxiety control group was coded as a reference group (coded as 000), while the specific math anxiety group (coded as 100), specific reading anxiety group (coded as 010), and specific trait anxiety group (coded as 001) were coded as experimental groups. The other variables in the second and third path models were the same as those in the first path model.

## Results

### Group Differences in Anxiety, RSA, and Behavioral Tasks

Descriptive statistics of demographical data, behavioral measures, and physiological measures of different groups are shown in Table 1. Group differences were significant in all self-reported anxiety scores. Individuals in specific math anxiety group reported higher math anxiety than those in specific trait anxiety group, specific reading anxiety group, and no-anxiety control group. Individuals in the specific trait anxiety group reported higher trait anxiety than those in the specific math anxiety group, specific reading anxiety group, and no-anxiety control group. Individuals in the specific reading anxiety group reported higher reading anxiety than those in the specific math anxiety group, specific trait anxiety group, and no-anxiety control group.

**Table 1:** Demographical data, behavioral scores and RSA reactivity in math and reading tasks of each group.

		**Control group**		**Math anxiety group**		**Trait anxiety group**		**Reading anxiety group**	**Test statistics**
	**N**	**Mean (SD)**	**N**	**Mean (SD)**	**N**	**Mean (SD)**	**N**	**Mean (SD)**	
**Subject characteristics**									
Gender (boy/girl)	22	14/8	29	15/14	24	14/10	29	19/10	χ^2^(3) = 1.33, *p* = 0.72
Age (months)	22	169.41 (11.89)	29	165.34 (6.43)	24	166.83 (8.07)	29	164.86 (9.08)	*F*_(3,100)_ = 1.28, *p* = 0.29, η^2^ = 0.04
**Anxiety scores**									
Math anxiety (21–105)	22	25.64 (4.04)	29	43.55 (5.18)	24	28.88 (5.19)	29	29.45 (4.51)	*F*_(3,100)_ = 75.43, *p* < 0.001, η^2^ = 0.69
Reading anxiety (20–100)	22	31.45 (3.94)	29	46.17 (6.52)	24	44.92 (7.63)	29	62.21 (8.45)	*F*_(3,100)_ = 83.37, *p* < 0.001, η^2^ = 0.71
Trait anxiety (20–80)	22	29.45 (2.77)	29	41.24 (3.54)	24	49.33 (3.56)	29	41.93 (3.35)	*F*_(3,100)_ = 137.82, *p* < 0.001, η^2^ = 0.81
**Math task**									
Accuracy	22	0.99 (0.01)	29	0.98 (0.01)	24	0.98 (0.02)	29	0.98 (0.02)	*F*_(3,100)_ = 1.09, *p* = 0.36, η^2^ = 0.03
Reaction time (/s)	22	1.18 (0.16)	29	1.38 (0.30)	24	1.26 (0.19)	29	1.20 (0.23)	*F*_(3,100)_ = 3.87, *p* = 0.01, η^2^ = 0.10
RSA reactivity (ms^2^)	22	0.11 (0.48)	29	−0.23 (0.67)	24	0.30 (0.46)	29	0.17 (0.41)	*F*_(3,100)_ = 5.33, *p* = 0.002, η^2^ = 0.14
**Reading task**									
Correct number (/195)	22	180.50 (6.05)	29	180.97 (7.62)	24	180.75 (6.50)	29	176.55 (8.83)	*F*_(3,100)_ = 2.24, *p* = 0.09, η^2^ = 0.06
Total naming time (/s)	22	203.27 (45.51)	29	225.21 (59.25)	24	206.33 (62.29)	29	217.93 (61.43)	*F*_(3,100)_ = 0.80, *p* = 0.50, η^2^ = 0.02
RSA reactivity (ms^2^)	22	−0.07 (0.32)	29	−0.11 (0.50)	24	0.11 (0.44)	29	0.01 (0.45)	*F*_(3,100)_ = 1.26, *p* = 0.29, η^2^ = 0.04

**Figure 1:**
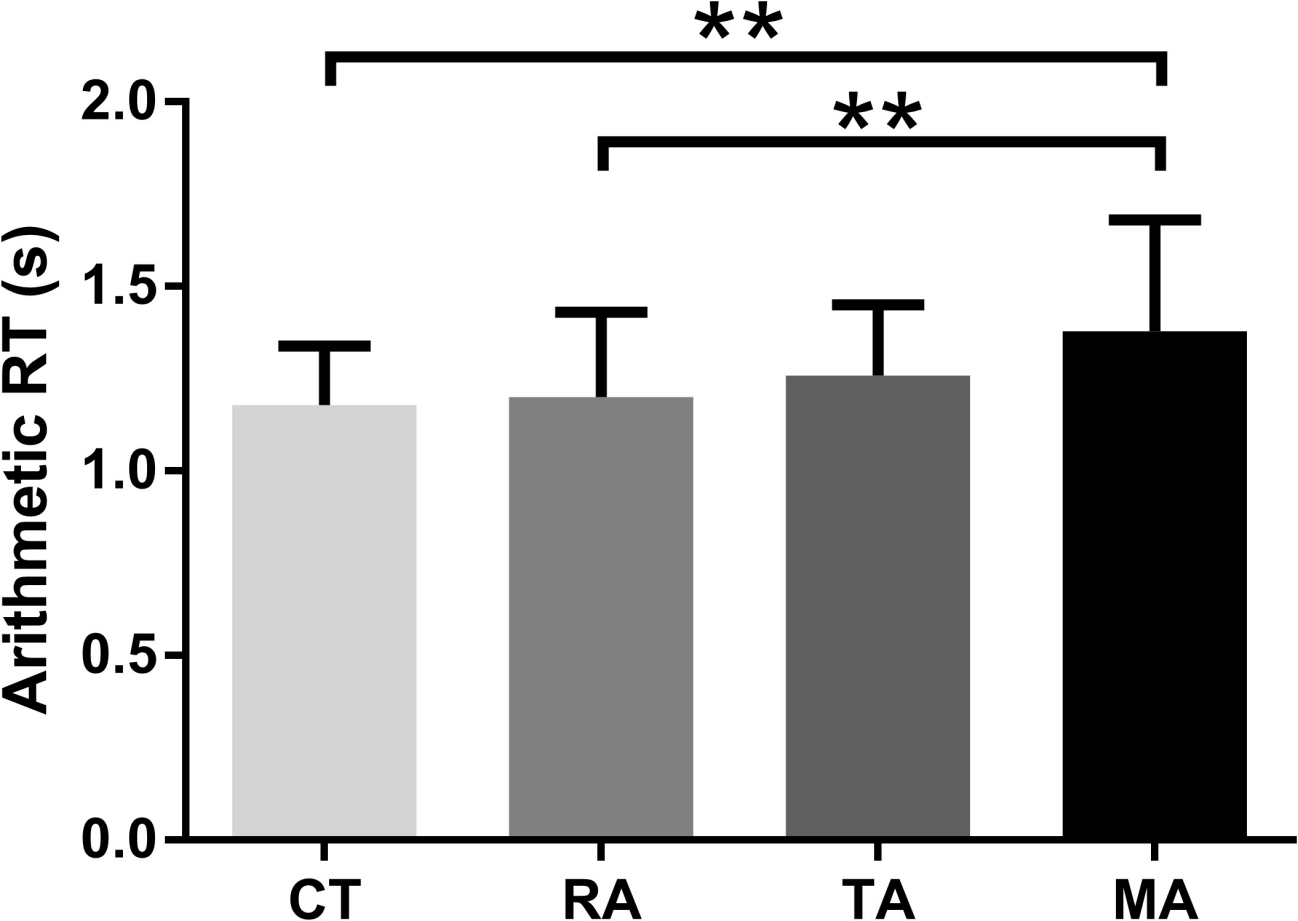
Mean (standard error) of reaction time (RT) in the arithmetic task. CT: control group, TA: trait anxiety group, MA, math anxiety group; RA, reading anxiety group. **p < 0.01.

**Figure 2:**
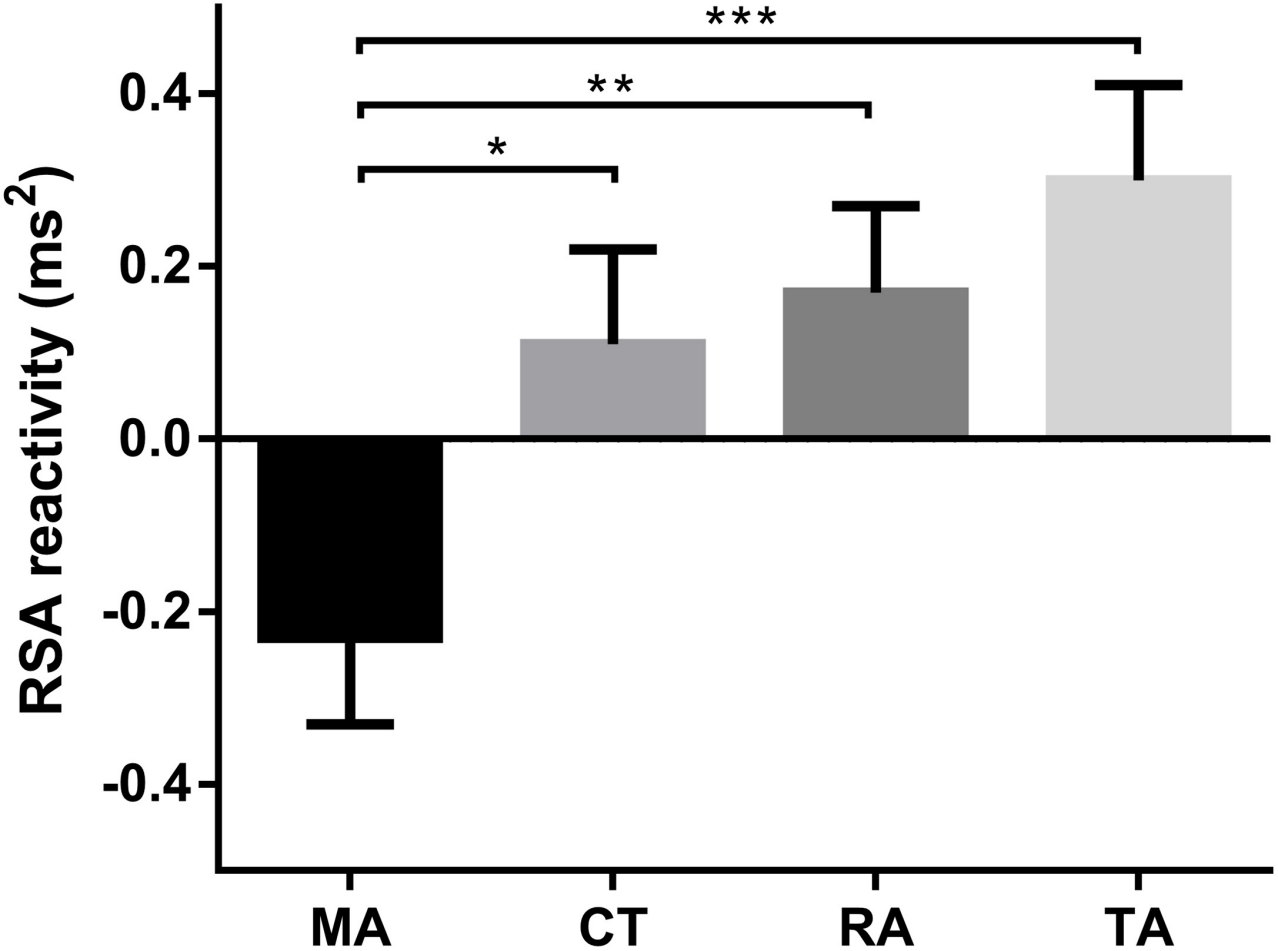
Mean (standard error) of respiratory sinus arrhythmia (RSA) reactivity in the arithmetic task. CT, control group; TA, trait anxiety group; MA, math anxiety group; RA, reading anxiety group. *p < 0.05, **p < 0.01, ***p < 0.001.

Significant group differences were found in arithmetic RT [*F*_(3,100)_ = 3.87, *p* = 0.01, η^2^ = 0.10). *Post-hoc* comparisons of RTs between different groups in the math task revealed that RT of the specific math anxiety group was longer than RTs of the other three groups (Figure 1; control group: Cohen's *d* = 0.83, *p* < 0.01, FDR *q* = 0.024; specific reading anxiety group: Cohen's *d* = 0.67, *p* < 0.01, FDR *q* = 0.015; specific trait anxiety group: Cohen's *d* = 0.48, *p* = 0.06, FDR *q* = 0.124). Significant group differences were also found in RSA reactivity in the arithmetic task [*F*_(3,100)_ = 5.33, *p* = 0.002, η^2^ = 0.14). *Post-hoc* comparisons of RSA reactivity between different groups revealed that RSA reactivity was significantly lower in the specific math anxiety group than in the other three groups (Figure 2; control group: Cohen's *d* = 0.58, *p* < 0.05, FDR *q* = 0.044; specific trait anxiety group: Cohen's *d* = 0.92, *p* < 0.001, FDR *q* = 0.0018; and specific reading anxiety group: Cohen's *d* = 0.72, *p* < 0.01, FDR *q* = 0.012). However, no group difference was found in RSA baseline or RSA in arithmetic tasks. No group difference was found in accuracy for the math task due to ceiling effect, and no group difference was found in any measurement of the reading task. No parental education difference or grade difference was found in any variable.

### Correlations Between Anxiety, RSA, and Behavioral Tasks

Correlations between anxiety scores, RSA, and performance of behavioral tasks are shown in Table 2. Results showed that self-reported math anxiety was significantly correlated with arithmetic RT (*r* = 0.275, *p* < 0.01) and RSA reactivity (*r* = −0.219, *p* < 0.05) in the arithmetic task, indicating higher math anxiety related to higher physiological anxiety and longer RT in the arithmetic task. RSA reactivity and RT also significantly correlated with each other (*r* = −0.330, *p* < 0.01). Though reading anxiety significantly correlated with reading accuracy (*r* = −0.208, *p* < 0.05), RSA reactivity in the reading task was not correlated with any of the reading measurements.

**Table 2:** Correlations of all measurements.

	**Trait anxiety**	**Math anxiety**	**Reading anxiety**	**Math RT**	**Math ACC**	**Reading RT**	**Reading CN**	**RSA in math task**	**RSA in reading task**
Trait anxiety	1								
Math anxiety	0.176	1							
Reading anxiety	0.390^***^	0.124	1						
Math ACC	0.099	0.275^**^	−0.014	1					
Math CR	−0.019	0.027	−0.068	0.032	1				
Reading RT	−0.017	0.133	0.092	0.333^**^	−0.068	1			
Reading CN	0.063	0.094	−0.208^*^	−0.202^*^	0.211^*^	−0.371^***^	1		
RSA in math task	0.136	−0.219^*^	0.147	−0.330^**^	−0.103	−0.245^*^	0.115	1	
RSA in reading task	−0.130	0.048	−0.080	0.073	0.244^*^	0.101	−0.058	−0.504^***^	1

*Math RT stands for average reaction time in math task. Math ACC stands for accuracy in math task. Reading RT stands for reaction time in reading task. Reading CN stands for correct character number in reading task*.

* *p < 0.05*,

** *p < 0.01*,

*** *p < 0.001*.

### Hierarchical Multiple-Regression Analyses

As shown in Table 3, hierarchical linear-regression analysis of arithmetic RT showed that the specific math anxiety membership (specific math anxiety group coded as 1 and non-specific math anxiety group coded as 0) could significantly predict arithmetic RT after gender and measures (correct character number, RT, and RSA) in reading tasks were regressed out. RSA reactivity could still significantly predict arithmetic RT after gender measures in reading tasks and group membership were regressed out.

**Table 3:** Hierarchical regression models of average arithmetic RT (reaction time) predicted by groups (specific math anxiety group and no specific math anxiety group) and RSA.

**Step**	**Predictors**	**Average arithmetic RT**	
		***R*^**2**^**	**β (SE)**
1	Gender	0.14^**^	0.15 (0.05)
	Correct character number		−0.10 (0.003)
	Reading reaction time		0.29 (0.0004)^**^
	RSA reactivity in reading task		0.04 (0.05)
2	Specific math anxiety group	0.21^***^	0.28 (0.05)^**^
3	RSA reactivity in arithmetic task	0.25^***^	−0.25 (0.05)^*^

* *p < 0.05*,

** *p < 0.01*,

*** *p < 0.001*.

### Mediation Analyses

**Figure 3:**
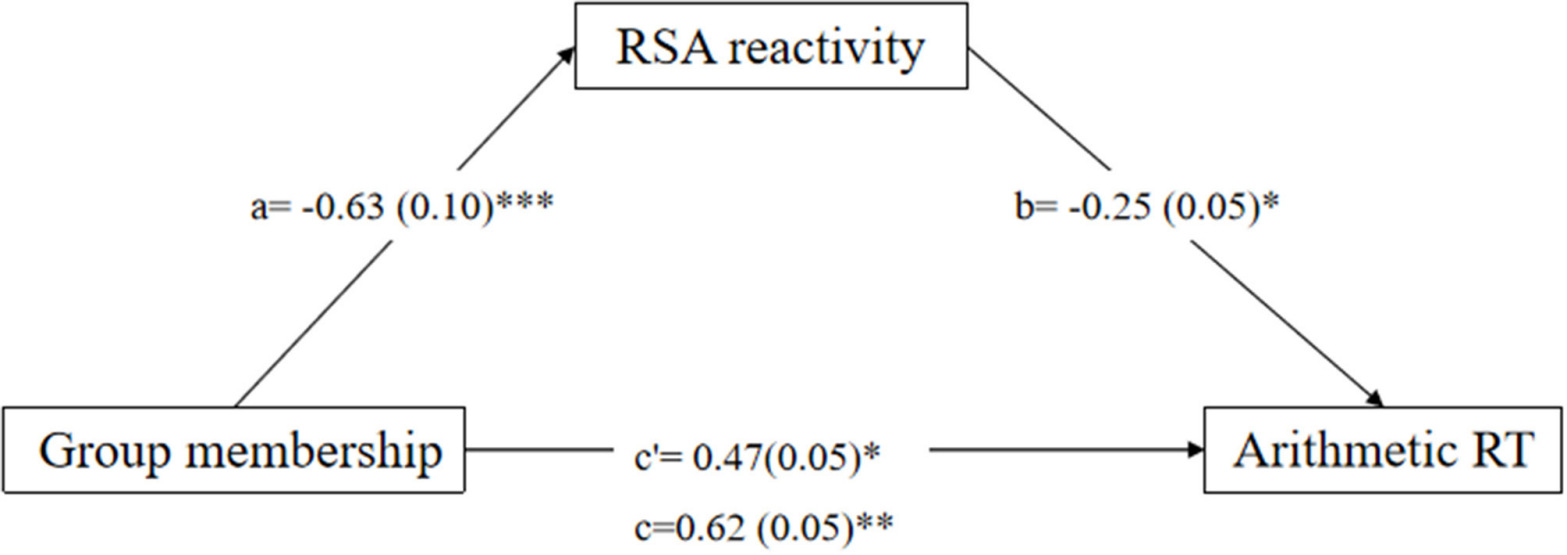
Mediation effect of respiratory sinus arrhythmia (RSA) reactivity on the relation between group memberships (specific math anxiety group and no specific math anxiety group) and reaction time (RT) in the arithmetic task. *p < 0.05, **p < 0.01, ***p < 0.001. One-headed arrows represent significant paths.

Firstly, specific math anxiety group membership (specific math anxiety group coded as 1 and non-specific math anxiety group coded as 0) was entered into the model as an independent variable. The mediation model of group membership → RSA reactivity → arithmetic RT fits the data well [*F*_(5,98)_ = 5.33; *p* < 0.001]. The mediation model (Figure 3) accounted for 25% of the variance in arithmetic RT. The total indirect effect accounted for 25% of the total effect. RSA reactivity mediates the relation between group membership and arithmetic RT, and the direct effect was significant (c = 0.62, *p* < 0.01, c′ = 0.47, *p* = 0.03, a ^*^ b = 0.15, 95% CI = [0.01, 0.32]).

**Figure 4:**
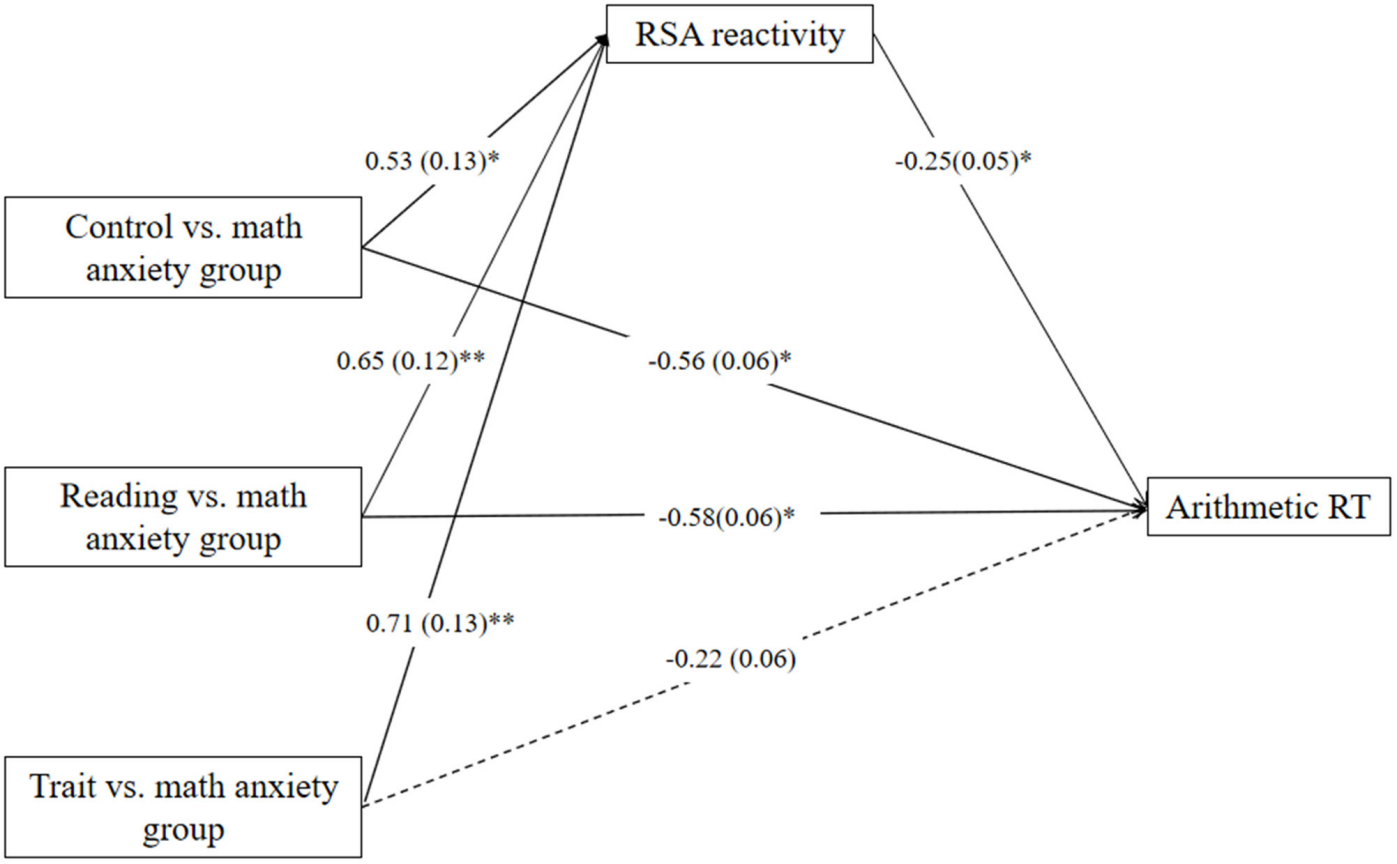
Mediation effect of respiratory sinus arrhythmia (RSA) reactivity on the relation between specific group membership and reaction time (RT) in the arithmetic task relative to specific math anxiety group. *p < 0.05, **p < 0.01. One-headed arrows represent significant.

The dummy variables were then entered into the model as independent variables. When the specific math anxiety group was coded as a reference category in the analysis, the mediation model of group membership → RSA reactivity → arithmetic RT also fits the data well [*F*_(7,96)_ = 4.10; *p* < 0.001]. The mediation model (Figure 4) accounted for 27% of the variance in arithmetic RT. The relative total indirect effect accounted for 26% of the relative total effect. RSA reactivity significantly mediates the relation between specific reading/trait anxiety group and arithmetic RT but marginally mediates the relation between control group and arithmetic RT relative to the specific math anxiety group (control group: c = −0.70, *p* < 0.01, c′ = −0.56, *p* = 0.03, a ^*^ b = −0.14, 95% CI = [−0.33, 0.002]; trait anxiety group: c = −0.40, *p* = 0.12, c′ = −0.22, *p* = 0.40, a ^*^ b = −0.18, 95% CI = [−0.37, −0.02]; reading anxiety group: c = −0.75, *p* < 0.01, c′ = −0.58, *p* = 0.02, a ^*^ b = −0.17, 95% CI = [−0.35, −0.02]).

**Figure 5:**
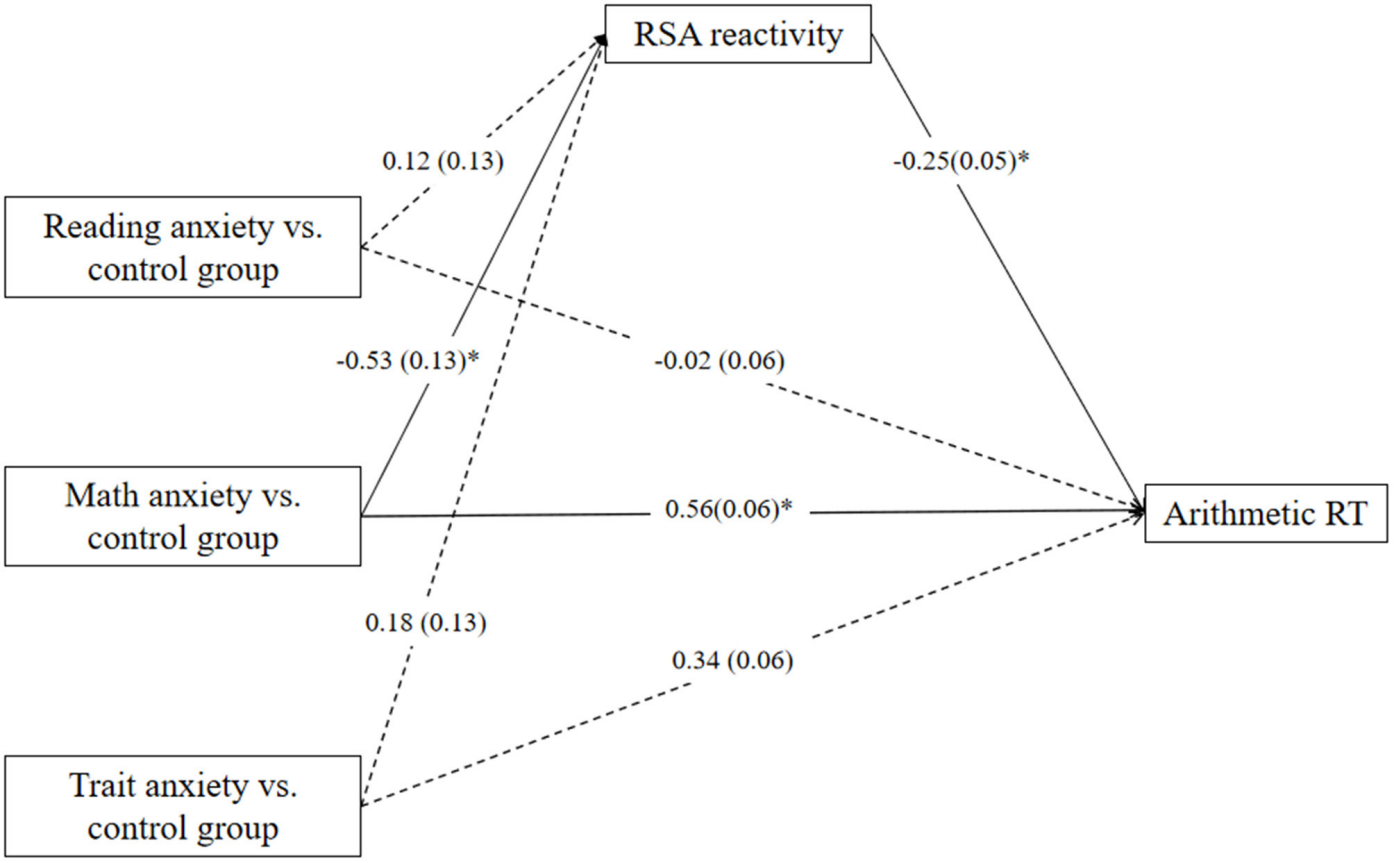
Mediation effect of respiratory sinus arrhythmia (RSA) reactivity on the relation between specific group membership and reaction time (RT) in the arithmetic task relative to control group. *p < 0.05. One-headed arrows represent significant paths.

When the no-anxiety control group was coded as a reference category in the analysis, the mediation model of group membership → RSA reactivity → arithmetic RT fits the data well [*F*_(7,96)_ = 4.10; *p* < 0.001]. The mediation model (Figure 5) accounted for 27% of the variance in arithmetic RT. The relative total indirect effect accounted for 6% of the relative total effect. RSA reactivity only marginally mediates the relation between specific math anxiety group and arithmetic RT relative to the control group, and the direct effect was significant (specific math anxiety group: c = 0.70, *p* < 0.01, c′ = 0.56, *p* = 0.03, a ^*^ b = 0.14, 95% CI = [−0.003, 0.33]; specific trait anxiety group: c = 0.30, *p* = 0.28, c′ = 0.34, *p* = 0.20, a ^*^ b = −0.04, 95% CI = [−0.19, 0.10]; specific reading anxiety group: c = −0.05, *p* = 0.85, c′ = −0.02, *p* = 0.94, a ^*^ b = −0.03, 95% CI = [−0.17, 0.10]).

## Discussion

In this study, we explored the relations between specific math anxiety, RSA, and arithmetic speed. We found that individuals with specific math anxiety tended to show larger RSA supression and longer RT in arithmetic tasks. Specifically, the relation between specific math anxiety and arithmetic RT was mediated by RSA reactivity. These results suggest a key role of the affective component of specific math anxiety in explaining the variance of math performance.

Firstly, the present study revealed that RT of the specific math anxiety group was longer than RTs of the non-specific math anxiety groups. By separating our participants into four groups (specific math anxiety, specific trait anxiety, specific reading anxiety group, and no-anxiety control group), our study isolated individuals with specific math anxiety and revealed longer RT in individuals with specific math anxiety when they completed arithmetic tasks. The results thus were not only consistent with previous findings which reported a negative correlation between math anxiety and math performance (see review by Carey et al., 2016) but also provide the first account of evidence for poor math performance in individuals with math anxiety due to math anxiety *per se* but not trait anxiety or reading anxiety. However, the math task we employed in the present study was a simple arithmetic task, which was relatively easy for middle school students. Therefore, we did not observe a difference in accuracy between specific math anxiety group and non-specific math anxiety group in our arithmetic tasks. Nevertheless, this does not mean that individuals with specific math anxiety are as accurate as individuals with non-specific math anxiety in all math tasks. Future studies measuring accuracy in difficult arithmetic tasks (e.g., three-digit numbers) and/or math problem-solving tasks might be valuable to be conducted in specific math anxiety.

Secondly, we also observed RSA suppression in the arithmetic tasks in middle school students with specific math anxiety, but not those without specific math anxiety. The RSA suppression, defined as the decreased RSA reactivity from baseline in cognitive resource-demanding tasks, reflects the withdrawal of vagal tone and an activation of the parasympathetic nervous system and is often used as an indicator of affective arousal (Frazier et al., 2004). Our results therefore suggest that students with specific math anxiety are likely to have higher affective responses in math-related situations than students without specific math anxiety. Our studies offered the first direct evidence for the affective account of specific math anxiety. Our results also provided complementary evidence to previous physiological study which observed a higher physiological/affective response level (e.g., cortisol, blood pressure, and heart rate) in math anxiety individuals when performing math tasks (Ushiyama et al., 1991; Mattarella-Micke et al., 2011; Hunt et al., 2017). Suppressed RSA in the specific math anxiety group was also broadly consistent with previous studies that have found suppressed RSA of individuals with anxiety disorders (e.g., Jovanovic et al., 2009). Although specific math anxiety individuals may also adapt to regulate their emotions in arithmetic tasks, longer RTs and RSA suppression of these individuals when performing arithmetic tasks in the present study suggest that their physiological reaction and behavioral performance would not be able to be compensated by emotional adaption (Beauchaine, 2001; Calkins and Keane, 2004; Gentzler et al., 2009).

Last but not least, the present study revealed that affective responses (as measured by RSA reactivity) mediated the relationship between specific math anxiety and arithmetic speed (as measured by RT). Our studies offered direct evidence for the key role of affective component of specific math anxiety in the explanation of math performance independent from trait anxiety and reading anxiety. Although previous studies have indicated that RSA represents one of the neural foundations for emotion and behavior and plays a linkage role between affective response and external behaviors (Porges, 2001), the present study provides the first account of evidence for the affective hypothesis of RSA in math anxiety and suggests that affective responses indexed by RSA reactivity may be an important neural mechanism underlying the detrimental effect of math anxiety on math performance. More importantly, these results provide experimental support for the independent role of RSA reactivity between specific math anxiety and math performance but not between trait/reading anxiety and math performance. Our results may thus suggest that only specific math anxiety initially trigger high physiological anxiety in mathematical situations, which in turn leads to poor math performance, although longitudinal or experimental studies might be required to further test this suggestion. It should be noted that the indirect effect of RSA reactivity in relating the specific math anxiety group and arithmetic speed was marginally significant relative to the no-anxiety control group. This may be due to a restricted number of participants in each group and large RSA variance among participants.

Admittedly, there are some limitations in the present study. Firstly, our results are merely correlational and cannot provide conclusions concerning the causal effect of affective math anxiety on math performance. Longitudinal or experimental studies would be needed to examine this issue. Secondly, we only adopted one-digit arithmetic tasks in the present study. It might be valuable to manipulate arithmetic task difficulty or examine other math problems with physiological responses recorded in individuals with various levels of math anxiety. Thirdly, a subsample of participants were selected to participate in the present study which restrained us from performing a mediation analysis with math anxiety as a continuous independent variable and draw a broader conclusion for the nature of math anxiety. Future studies would be valuable to collect RSA from the whole sample and should conduct mediation analysis in the whole sample, in order to provide a more comprehensive insight into the role of RSA in the relation between math anxiety and arithmetic speed. As math anxiety of the participants in the subsample of the current study still followed a normal distribution (see Supplementary Table S1), we ran a mediation model with math anxiety as a continuous independent variable as a complement, and results revealed a mediation effect of RSA between math anxiety and arithmetic speed in the current subsample (see Supplementary Figure S1). Finally, there is possibility that other affective and physiological mediators, such as cortisol, exist to explain the individual difference in math achievement. Future studies including other physiological measurements can further investigate this issue.

## Conclusions

In conclusion, this research shows that individuals with specific math anxiety respond slower and have larger physiological response indexed by RSA supression in arithmetic tasks. Specific math anxiety predicts arithmetic speed via RSA supression. Our results support an affective explanation for the relation between specific math anxiety and arithmetic speed. These results thus provide the first insight into the physiological and affective mechanisms underlying the association between specific math anxiety and math performance. Our findings are also of interest for educators, indicating the importance of helping students relieve and cope with specific math anxiety in order to improve their math achievement.

## Data Availability Statement

The raw data supporting the conclusions of this article will be made available by the authors, without undue reservation.

## Ethics Statement

The studies involving human participants were reviewed and approved by Committee on Human Research Protection of Shaanxi Normal University. Written informed consent to participate in this study was provided by the participants' legal guardian/next of kin.

## Author Contributions

JT performed the analysis and wrote the manuscript. YS designed the study, collected the data and performed the analysis. YY performed the analysis. HP performed the analysis and revised the manuscript. AG revised the manuscript. JZ designed the study, performed the analysis and wrote the manuscript. All authors contributed to the article and approved the submitted version.

## Conflict of Interest

The authors declare that the research was conducted in the absence of any commercial or financial relationships that could be construed as a potential conflict of interest.
