# Supplementary material for: Respiratory Sinus Arrhythmia Mediates the Relation Between “Specific Math Anxiety” and Arithmetic Speed
Source: Front Psychol. 2021 Feb 19;12:615601. doi: 10.3389/fpsyg.2021.615601 (PMC7933226; doi:10.3389/fpsyg.2021.615601)
Supplement: Supplementary file 1 [file Data_Sheet_1.docx]

**Supplementary materials**

Table S1. Distribution of math anxiety, trait anxiety and reading anxiety in the sub-sample.

|  | Skewness | | Kurtosis | |
| --- | --- | --- | --- | --- |
|  | N | Mean (SD) | N | Mean (SD) |
| Math anxiety (21-105) | 104 | 0.66 (0.24) | 104 | -0.05 (0.47) |
| Reading anxiety (20-100) | 104 | 0.39 (0.24) | 104 | 0.40 (0.47) |
| Trait anxiety (20-80) | 104 | -0.31 (0.24) | 104 | -0.27 (0.47) |

|  | Table S2. Demographical data, anxiety scores of the whole sample (N=386). | | | | | | | | | |  |
| --- | --- | --- | --- | --- | --- | --- | --- | --- | --- | --- | --- |
|  | | Boys | | | |  | Girls | | | |  |
|  | | N | Mean (SD) | Skewness | Kurtosis |  | N | Mean (SD) | Skewness | Kurtosis | Test statistics |
| *Subject characteristics* | |  |  |  |  |  |  |  |  |  |  |
| Grade (7th/8th) | | 223 | 139/84 |  |  |  | 163 | 96/67 |  |  | *χ²* (1) = 0.47 ,*p* = 0.494 |
| Age (months) | | 223 | 169.05 (10.29) | 0.62 | -0.53 |  | 163 | 168.75(9.95) | 0.84 | 0.14 | *t* (384) = 0.30, *p* =0.775 |
| Maternal education (1-8) | | 223 | 3.27 (1.89) | -0.62 | -0.86 |  | 163 | 3.07 (2.12) | -0.32 | -1.13 | *t* (384) = 0.99, *p* =0.324 |
| Paternal education (1-8) | | 223 | 3.42 (2.00) | -0.63 | -0.78 |  | 163 | 3.28(2.22) | -0.44 | -1.17 | *t* (384) = 0.64, *p* =0.521 |
| *Anxiety scores* | |  |  |  |  |  |  |  |  |  |  |
| Math anxiety (21-105) | | 223 | 30.86(9.28) | 1.67 | 3.87 |  | 163 | 36.04 (12.00) | 1.51 | 3.19 | *t* (384) = -4.60, *p*< 0.001 |
| Reading anxiety (20-100) | | 223 | 47.36 (12.13) | 0.35 | 0.14 |  | 163 | 47.12 (11.80) | 0.30 | -0.19 | *t* (384) = 0.20, *p* = 0.842 |
| Trait anxiety (20-80) | | 223 | 41.48(7.73) | 0.37 | 0.40 |  | 163 | 43.74 (7.78) | 0.25 | -0.30 | *t* (384) = -2.83, *p* = 0.005 |

**
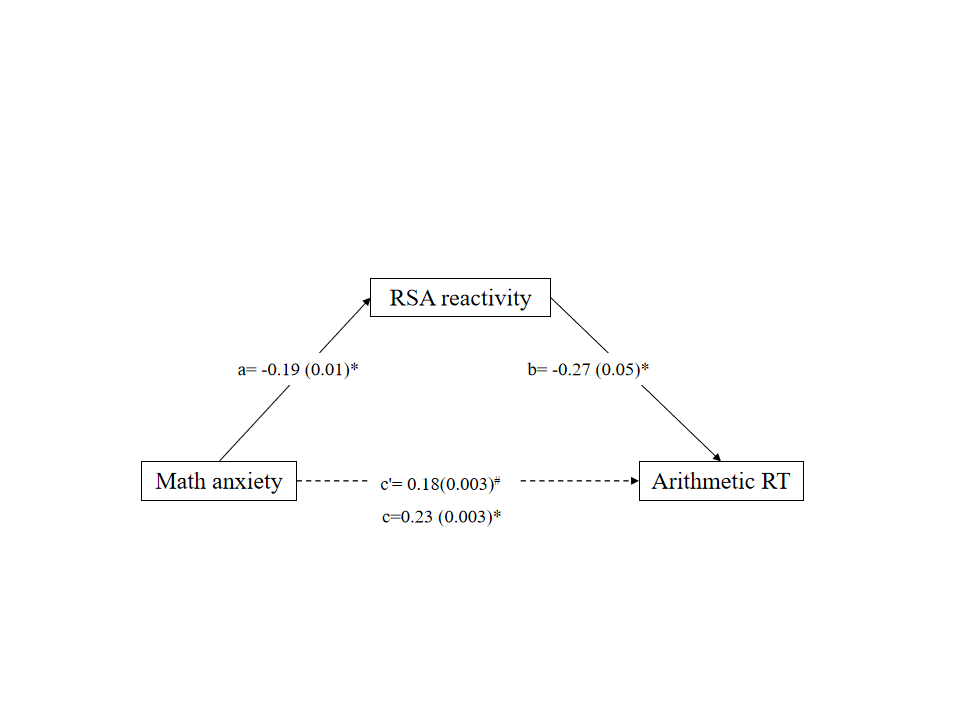
**

**Figure S1.** Mediation effect of RSA reactivity on the relation between math anxiety and reaction time (RT) in the arithmetic task. *^#^p*< .1, * *p*< .05. One-headed arrows represent significant paths.
